# Investigation of N_3_C_5_ and B_3_C_5_ bilayers as anode materials for Li-ion batteries by first-principles calculations

**DOI:** 10.1038/s41598-024-61939-x

**Published:** 2024-05-16

**Authors:** Grzegorz T. Kasprzak, Marcin W. Jarosik, Artur P. Durajski

**Affiliations:** Institute of Physics, Czestochowa University of Technology, Ave. Armii Krajowej 19, 42-200 Czestochowa, Poland

**Keywords:** Graphene, Electronic properties, Anode material, DFT calculations, Electronic properties and materials, Electronic properties and devices

## Abstract

The best choice today for a realistic method of increasing the energy density of a metal-ion battery is to find novel, effective electrode materials. In this paper, we present a theoretical investigation of the properties of hitherto unreported two-dimensional B_3_C_5_ and N_3_C_5_ bilayer systems as potential anode materials for lithium-ion batteries. The simulation results show that N_3_C_5_ bilayer is not suitable for anode material due to its thermal instability. On the other hand B_3_C_5_ is stable, has good electrical conductivity, and is intrinsically metallic before and after lithium intercalation. The low diffusion barrier (0.27 eV) of Li atoms shows a good charge and discharge rate for B_3_C_5_ bilayer. Moreover, the high theoretical specific capacity (579.57 mAh/g) connected with moderate volume expansion effect during charge/discharge processes indicates that B_3_C_5_ is a promising anode material for Li-ion batteries.

## Introduction

Studies into alternative energy sources, particularly renewable energy, are being driven by the modern world’s rising energy consumption, concerns about the fossil fuel dilemma, and the need for environmental protection^1^. The method of storing the generated energy is also a huge challenge. Currently, lithium-ion batteries (LIBs) are the preferred energy storage technology for various applications, including portable electronics, electric vehicles, and grid energy storage^2,3^. The materials in use as electrodes have a significant impact on the properties of LIBs. In industrial LIBs, graphite is the anode material that is most frequently used^4,5^. According to estimates, graphite has a theoretical capacity of 372 mAh/g^6^. Unfortunately, the main drawbacks of the graphite electrode, which restrict its use and advancement, are structural deformation, initial loss of capacity, and electrical disconnect^7^. As one of the alternatives to the commonly used graphite as an anode material, titanium dioxide (TiO_2_) has been looked into. The attractive properties of TiO_2_, which include low cost, high chemical stability, low volume expansion during charging/discharging, eco-friendliness, high energy density, and ease of availability, have made it one of the promising anode materials for LIBs^8,9^. Unfortunately, in comparison with commercially used graphite anode materials, TiO_2_ has a relatively low theoretical capacity of around 335 mAh/g^10^.

The continuous demand for higher energy density, improved cycling stability, and longer battery life drives extensive research on developing advanced materials for LIB electrodes. In this context, two-dimensional (2D) materials have emerged as promising candidates for enhancing the performance of LIB anodes.

The unique properties of 2D materials, such as large surface area, atomically thin structure, and tunable electronic properties, make them attractive for energy storage applications^11–16^. The atomically thin nature of 2D materials allows for efficient lithium ion diffusion and accommodation during charge and discharge processes, leading to improved capacity and cycling stability. Additionally, the large surface area of 2D materials facilitates increased electrochemically active sites, enhancing the overall electrochemical performance of the anode^17^. Several types of 2D materials have been recently investigated as potential anode materials for LIBs. These include transition metal dichalcogenides (TMDs) such as MoS_2_, graphene, MXenes, and various other 2D carbides, nitrides, and oxides. Each of these materials possesses unique properties that can influence their electrochemical behavior, capacity, and cycling stability^18–20^.

In the present study, we propose that graphene bilayers substitutionally doped with B and N atoms, specifically the B_3_C_5_ and N_3_C_5_ systems, are promising as potential anode materials for LIBs. Li-ion battery swelling during the intercalation of Li into the electrodes is a major cause of battery electrode degradation and can contribute significantly to cell failure. The swelling effect can only be investigated by studying the bilayer or bulk systems, therefore, herein we take into account bilayer systems. This investigation is based on DFT computations. It has been observed that both hitherto unreported B_3_C_5_ and N_3_C_5_ exhibit metallic properties with significant conductance after the adsorption of metal ions. Additionally, these systems demonstrate a remarkable specific Li capacity, which is significantly greater than that of pure graphene. Moreover, B_3_C_5_ and N_3_C_5_ display a low diffusion barrier and moderate volume expansion effect during charge/discharge processes. These attributes suggest that the investigated B_3_C_5_ and N_3_C_5_ systems could be ideal electrode materials for Li-ion batteries. Our comprehensive analysis aims to illuminate the potential of 2D materials as anode components for LIBs, providing insights into their distinctive properties and their contribution to the overall performance of lithium-ion batteries. The outcomes of this study have the potential to guide the design and development of advanced 2D anode materials, enhancing their electrochemical characteristics and elevating their energy storage capabilities.

## Computational methods

The first-principles calculations in this study were performed using the Quantum ESPRESSO package^21,22^, employing projector augmented wave (PAW) potentials within density-functional theory (DFT). The exchange-correlation energy of the electrons was treated within the generalized gradient approximation (GGA) functional of Perdew–Burke–Erzenhoff (PBE). The charge density and kinetic energy cut-off values were set to 500 Ry and 50 Ry, respectively. To minimize interactions between neighboring slabs, a vacuum of 20 Å was included. Van der Waals (VdW) interactions between the layers were accounted for using Grimme’s DFT-D correction^23^. The geometrical structure is optimized by using the Broyden-Fletcher-Goldfarb-Shanno (BFGS) quasi-Newton algorithm^24^ with the convergence criterion of $10^{-5}$ eV for energy. The forces on all atoms are less than 0.001 eV/Å. Structural optimization and band structure computations were performed using a Monkhorst pack k-point grid of $36 \times 36 \times 1$. The diffusion barrier is calculated by the climbing image nudged elastic band (Cl-NEB) method^25^. The thermal stability of the B_3_C_5_ and N_3_C_5_ bilayer systems was examined through ab initio molecular dynamics (AIMD) simulations at 300 K using the CP2K software^26^ with a constant volume and temperature ensemble (NVT ensemble) and a time step of 1 fs and 10000 ionic steps.

The intercalation energy of Li between B_3_C_5_ and N_3_C_5_ bilayers was computed as below:

1 $$\begin{aligned} E_{\textrm{int}}=({E_{\textrm{Li}_n{\textrm{X}_3C_5}}} - E_{\textrm{X}_3C_5} - nE_{\textrm{Li}})/n, \end{aligned}$$

where $E_{\textrm{X}_3C_5}$ is the total energy of pristine ${\textrm{X}_3C_5}$ bilayer (X = B or N), $E_{\textrm{Li}}$ is the energy of a single Li atom (in bulk *bcc* phase), ${E_{\textrm{Li}_n{\textrm{X}_3C_5}}}$ means the total energy of Li-intercalated ${\textrm{X}_3C_5}$ bilayer and *n* is the number of intercalated lithium atoms. From this definition, the more negative the value of $E_{\textrm{int}}$, the more it illustrates the exothermic nature of the reaction, indicating a higher likelihood of the reaction occurring. Conversely, a positive value of $E_{\textrm{int}}$ indicates an endothermic reaction, suggesting that the reaction is less likely to occur.

Theoretical Li capacity can be estimated as below:

2 $$\begin{aligned} C=\frac{nzF}{\textrm{M}_{X_3C_5}}, \end{aligned}$$

where *n* is the number of intercalated Li atoms ($n=16$), *z* is the valence number ($z=1$ for Li), *F* is the Faraday constant (26801 mAh/mol), and $\textrm{M}_{X_3C_5}$ is the molar mass of B_3_C_5_ or N_3_C_5_ bilayer.

## Results and discussion

**Figure 1:**
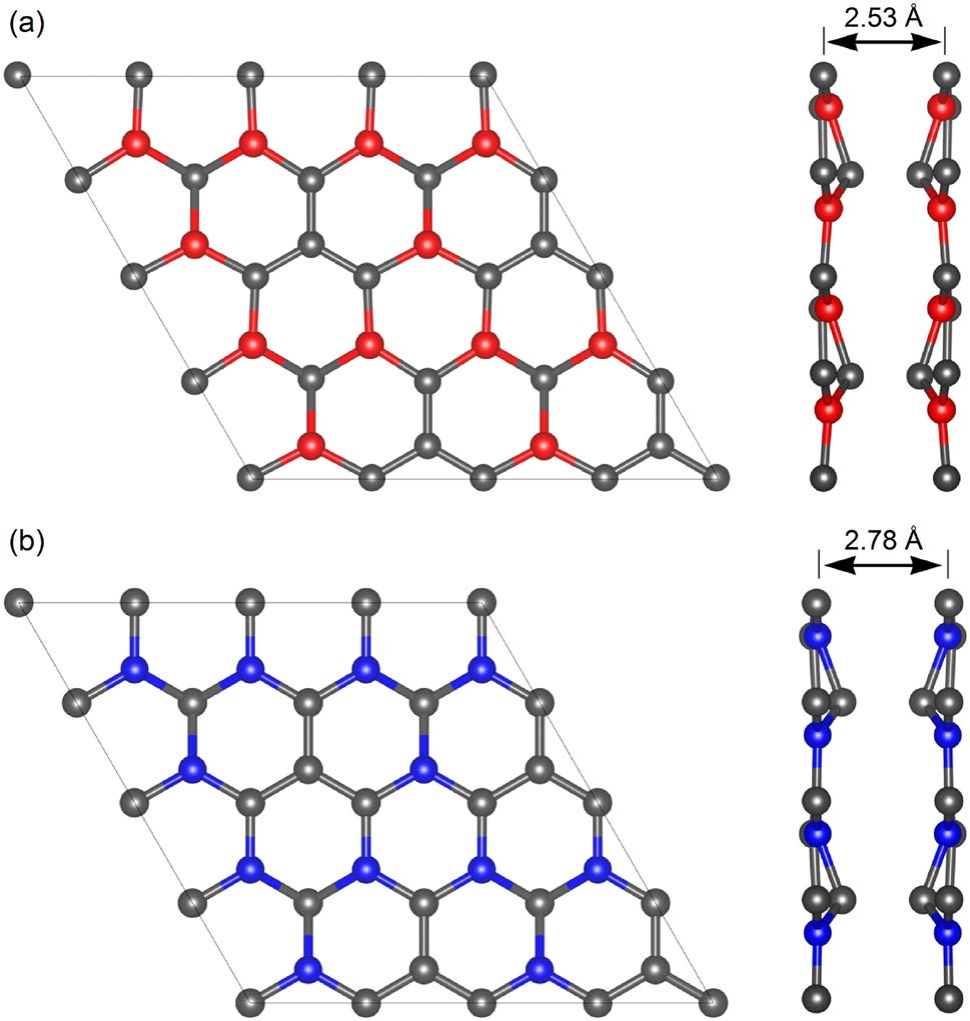
Atomic structure (top and side views) of pristine $2\times 2$ supercell of (**a**) B_3_C_5_ and (**b**) N_3_C_5_ bilayer.

A fully-relaxed structure of pristine B_3_C_5_ and N_3_C_5_ bilayer systems that have a hexagonal resemblance to graphene, but with a rippled surface, can be observed in Fig. 1a, b, respectively. Lattice constants *a* and *b* are both 5.22 Å for B_3_C_5_ and 4.81 Å for N_3_C_5_. The C–C and C–B bond lengths are 1.443 Å and 1.541 Å (1.592 Å) in the case of B_3_C_5_ and the C–C and C–N bond lengths are 1.387 Å and 1.388 Å (1.481 Å) in the case of N_3_C_5_.

To check the energetical stability of our bilayer structures, we evaluate the cohesive energies:

3 $$\begin{aligned} E_{\textrm{coh}} = \frac{6E_{\textrm{X}} + 10E_{\textrm{C}} - E_{\textrm{X}_3C_5}}{16}, \end{aligned}$$

where $E_{\textrm{X}}$ is the energy of an isolated B or N atom, $E_{\textrm{C}}$ means the energy of an isolated C atom, and $E_{\textrm{X}_3C_5}$ represents the total energy of X_3_C_5_ bilayer. The calculated cohesive energies of B_3_C_5_ and N_3_C_5_ bilayers are 7.76 eV/atom and 8.26 eV/atom, respectively. The higher cohesive energy indicates a more stable structure, indicating that the N_3_C_5_ monolayer has a more stable structure. The values of B_3_C_5_ and N_3_C_5_ monolayers are higher than those of phosphorene (3.48 eV/atom), silicene (3.71 eV/atom), SnC (5.5 eV/atom), GeP_3_ (3.34 eV/atom), P_3_C (4.18 eV/atom), MoS_2_ (4.98 eV/atom) and Mo_2_C (6.31 eV/atom) and close to Ti_3_BN monolayer (7.46 eV/atom) and graphene (7.95 eV/atom)^27–33^.

**Figure 2:**
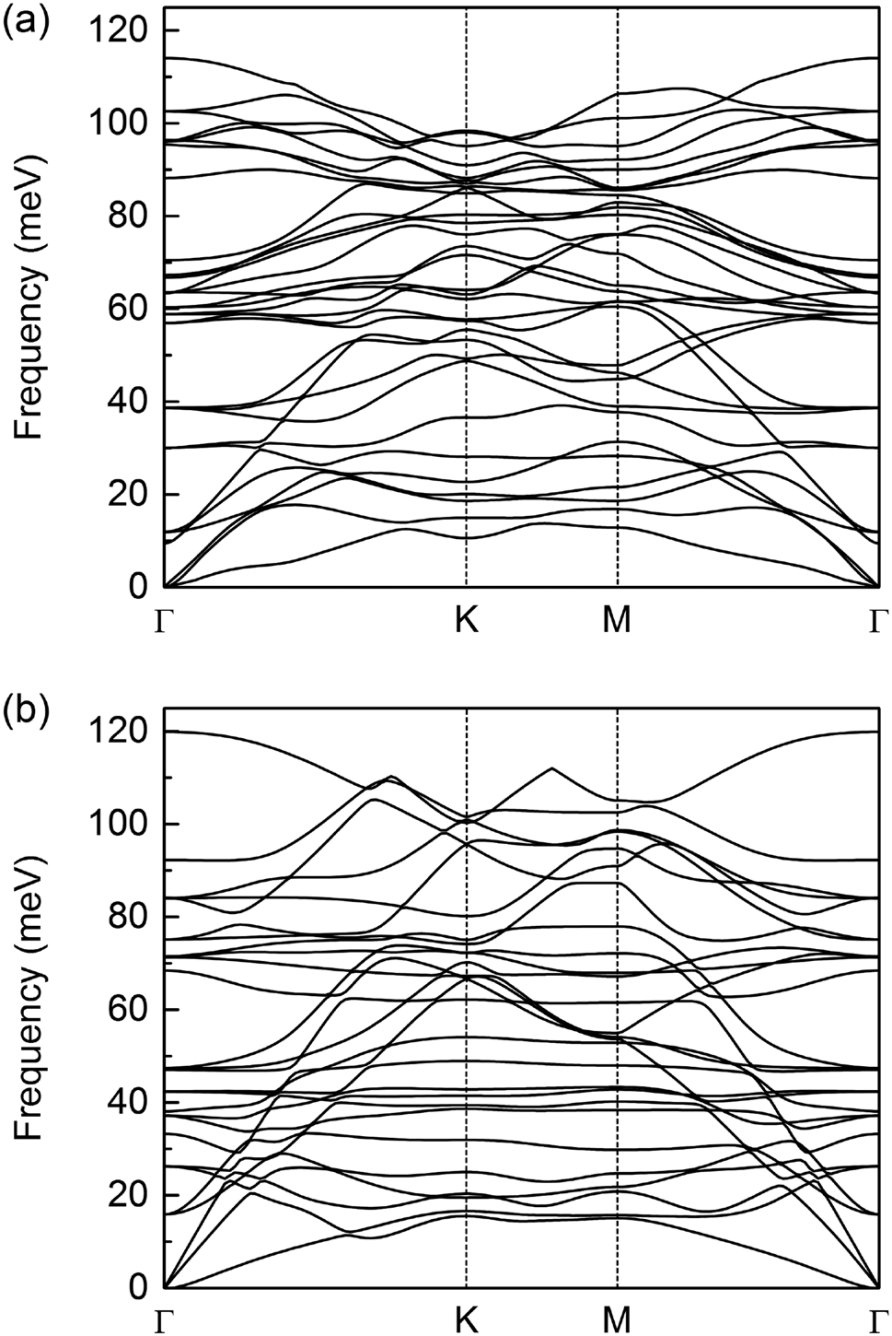
Calculated phonon spectra of (**a**) B_3_C_5_ bilayer and (**b**) N_3_C_5_ bilayer.

To investigate the dynamical stability of studied structures, phonon calculations were performed along $\Gamma$–M–K–$\Gamma$ symmetry points. As we can see in Fig. 2, no imaginary frequencies are observed in the entire Brillouin zone, confirming the dynamical stability of both investigated systems, which is an important result, especially from the energy storage point of view.

**Figure 3:**
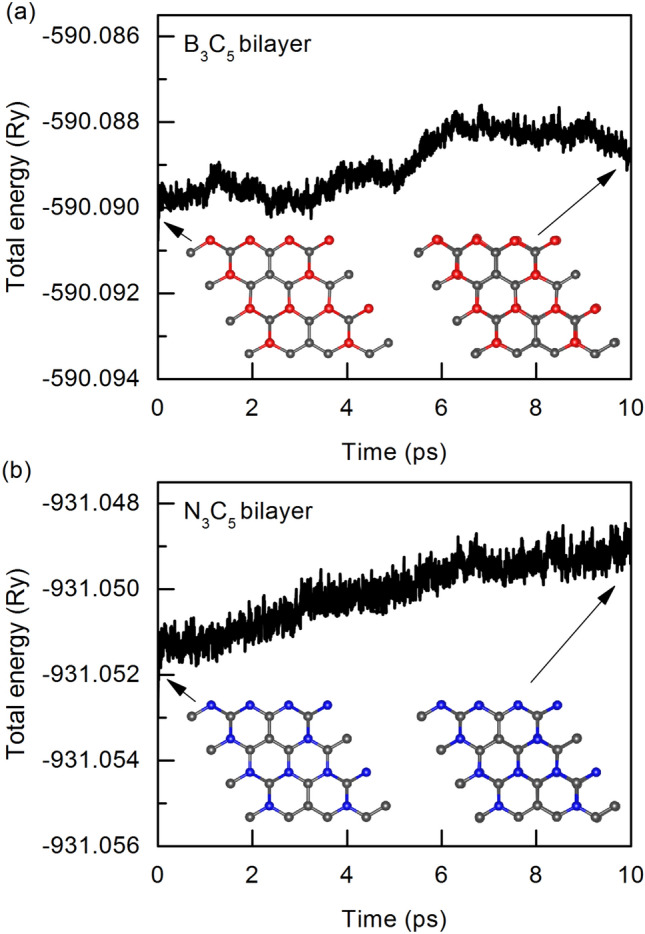
Fluctuation graphs of the total energy of $2\times 2$ (**a**) B_3_C_5_ and (**b**) N_3_C_5_ bilayers supercells as a function of time at 300 K. The embedded figures show the corresponding top views of the initial configurations and configurations after 10 ps of simulation.

We also used AIMD simulations to investigate the thermal stability of the B_3_C_5_ and N_3_C_5_ bilayers. Our structures retain their integrity after being heated to 300 K (see insert of Fig. 3). Simultaneously, the energy fluctuation is quite small, with a variation of around 0.003 Ry, indicating that both systems are thermally stable.

The intercalation energy ($E_{\textrm{int}}$) of B_3_C_5_ and N_3_C_5_ bilayers was estimated using Eq. (4). To denote the concentration of Li per unit bilayer cell, we use the notation of Li_n_(X_3_C_5_)_2_. The calculated $E_{\textrm{int}}$ of the single lithium atom (1:17 ratio) equals $-3.96$ eV and $-1.75$ eV for B_3_C_5_ and N_3_C_5_ bilayers, respectively. With further increase of Li number, the intercalation energy almost linearly decreases in the case of B_3_C_5_ and nonlinearly changes in the case of N_3_C_5_, as shown in Table 1. The obtained results showed that from the energy storage point of view, the B_3_C_5_ has better properties than the N_3_C_5_ due to the enhancement of intercalation energy. In the case of N_3_C_5_, the most energetically favorable is Li_3_(N_3_C_5_)_2_ configuration with the intercalation energy of $-2.46$ eV. The bilayer B_3_C_5_ exhibits an intercalation energy of $-4.43$ eV in the fully lithiated case, Li_4_(X_3_C_5_)_2_, illustrating that it is most promising and worth considering for the study of new anode materials for LIBs.

The formation energy relative to stable reference materials is crucial for assessing the stability of various phases at 0 K. The formation energy of structure with intermediate lithium content can be delineated as follows^34^:

4 $$\begin{aligned} E_{\textrm{f}}=\frac{{E_{\textrm{Li}_n{\textrm{X}_3C_5}}} - E_{\textrm{X}_3C_5} - nE_{\textrm{Li}}}{n+1}, \end{aligned}$$

**Figure 4:**
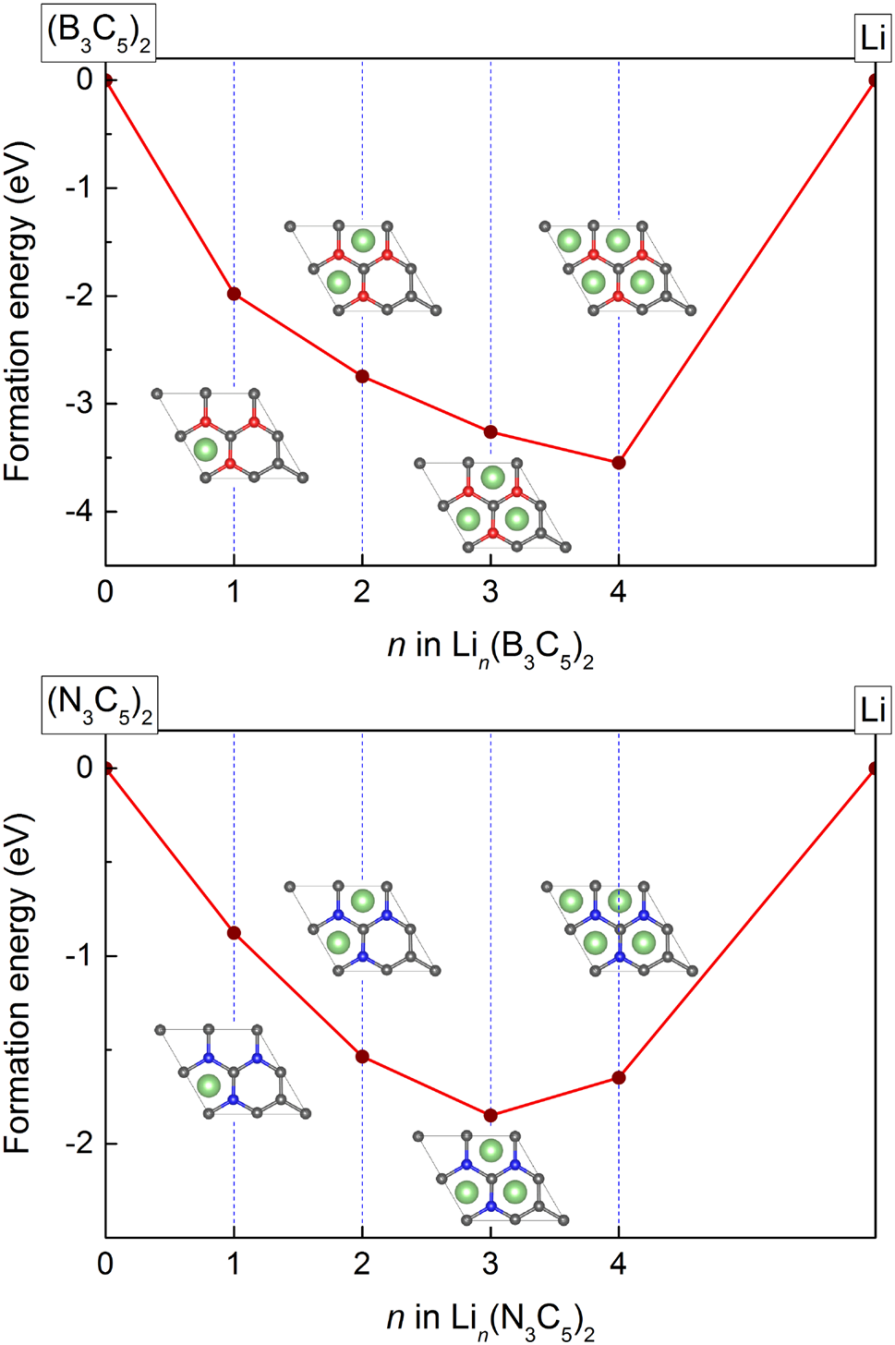
Formation energy convex hull of Li_n_(B_3_C_5_)_2_ and Li_n_(N_3_C_5_)_2_ systems.

The convex hulls obtained from the formation energies are presented in Fig. 4. The minimum value of $E_{\textrm{f}}$ corresponds to the most thermodynamically stable adsorption concentrations. The corresponding structures are illustrated in Fig. 4 insets.

It’s worth noting that a geometry optimization was conducted for different intercalation sites. The most energetically favorable position is found to be the hole position, where atoms from the initial bridge and top positions migrate during the optimization process. This indicates that the hole position is indeed the most energetically favorable configuration. Crystal structures of all Li-intercalated stages for Li_n_(B_3_C_5_)_2_ and Li_n_(N_3_C_5_)_2_ are included in Supplementary Information.

**Table 1:** Intercalation energy per lithium atoms (in eV) for the Li-intercalation between B_3_C_5_ and N_3_C_5_ bilayers as a function of Li atoms concentration in the unit cell.

n	Li_n_(B_3_C_5_)_2_	Li_n_(N_3_C_5_)_2_
1	$-3.96$	$-1.75$
2	$-4.12$	$-2.30$
3	$-4.35$	$-2.46$
4	$-4.43$	$-2.06$

**Figure 5:**
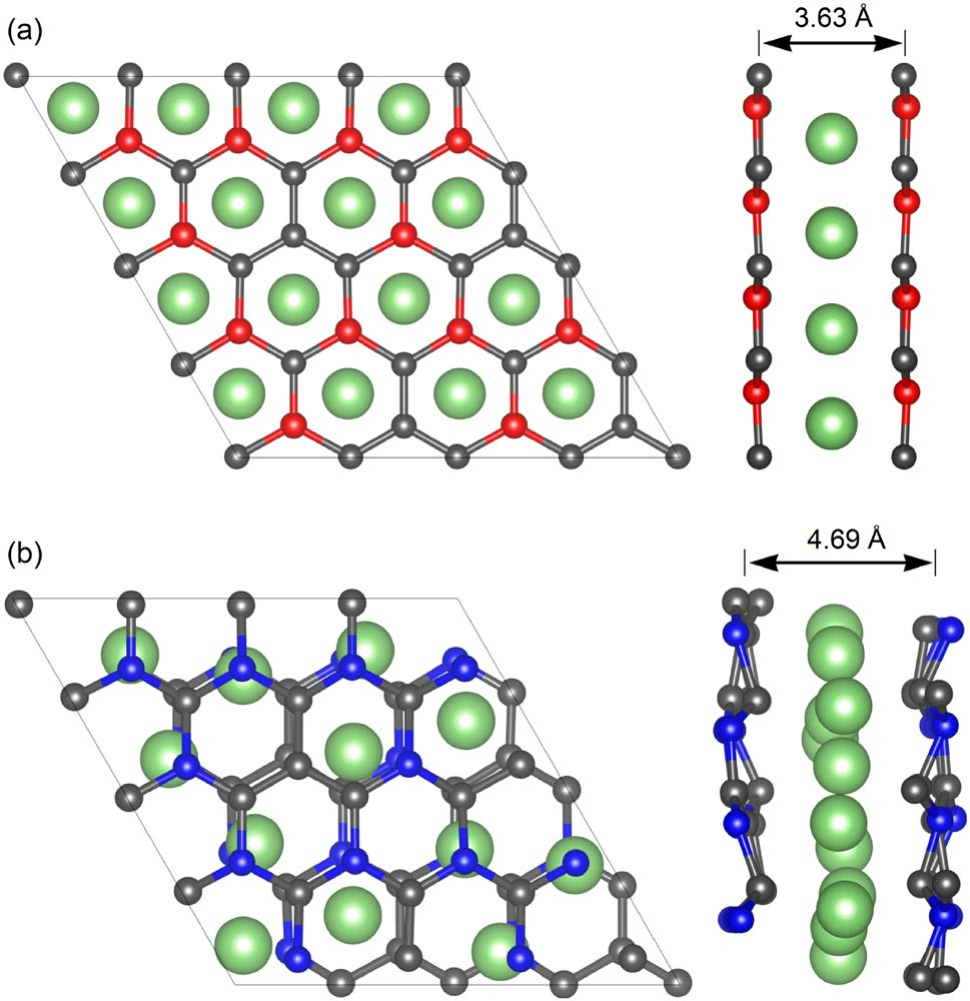
Atomic structure (top and side views) of Li-intercalated $2\times 2$ supercell of (**a**) B_3_C_5_ and (**b**) N_3_C_5_ bilayer.

After full lithiation (Li atoms intercalation between monolayers at all hole positions, Li_4_(B_3_C_5_)_2_), the monolayers in pristine B_3_C_5_ bilayer transforms from the initial structure with a rippled surface into a graphene-like plane structure, as shown in Fig. 5a. In the case of N_3_C_5_ bilayer (Li_3_(N_3_C_5_)_2_), the Li-intercalation contributes to the increase of rippling of the surface and changes ideal AA stacking where atoms of both layers have identical lateral coordinates into degenerate AA stacking where the one layer is slightly shifted relative to the other one (see Fig. 5b). Moreover, in both systems, the Li-intercalation increases the interlayer distance from 2.53 Å to 3.63 Å for B_3_C_5_ and from 2.78 Å to 4.69 Å in the case of N_3_C_5_. The practical application of new 2D anode materials is strongly impeded by large volume expansion during lithiation/delithiation processes which can result in loss of electrical contact with the conductive additive or the current collector and even peeling off from the current collector^35^. The results obtained for B_3_C_5_ and N_3_C_5_ clearly show a low volume expansion of about $43-69$%, compared to 2D Si/C composite (54%), commercial Si (183%) and pure Si nanosheets (95%)^36,37^.

**Figure 6:**
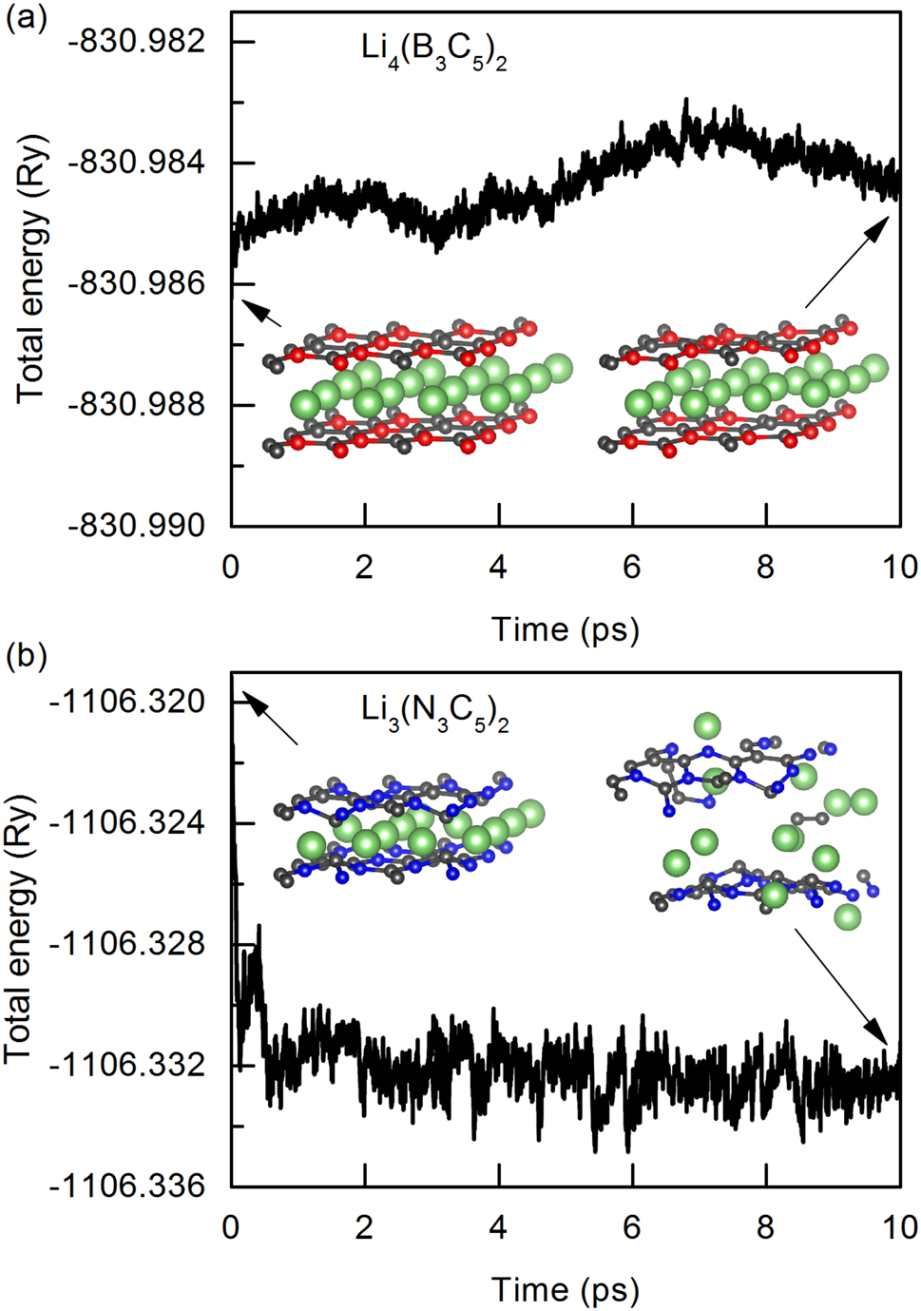
Fluctuation graphs of the total energy of $2\times 2$ (**a**) Li_4_(B_3_C_5_)_2_ and (**b**) Li_3_(N_3_C_5_)_2_ supercells as a function of time at 300 K. The embedded figures show the corresponding top views of the initial configurations and configurations after 10 ps of simulation.

Structural stability during the charging and discharging process at finite temperatures is an important factor in the performance of LIBs. An AIMD simulation is used to investigate the thermal stability of the Li_4_(B_3_C_5_)_2_ and Li_3_(N_3_C_5_)_2_ structures. Figure 6 show the calculated total energy of the fully lithiated bilayers during the AIMD simulation time of 10 ps at 300 K. As expected, the total energy of Li_4_(B_3_C_5_)_2_ does not change much during the AIMD steps. Such a slight change in energy indicates good thermal stability of a fully Li-intercalated B_3_C_5_ bilayer. In the case of Li_3_(N_3_C_5_)_2_ we observed structural deformation and energy drift, indicating that N_3_C_5_ bilayer is thermally unstable. Due to the above, we eliminated it from further analysis and we focused our attention only on the B_3_C_5_ bilayer.

**Figure 7:**
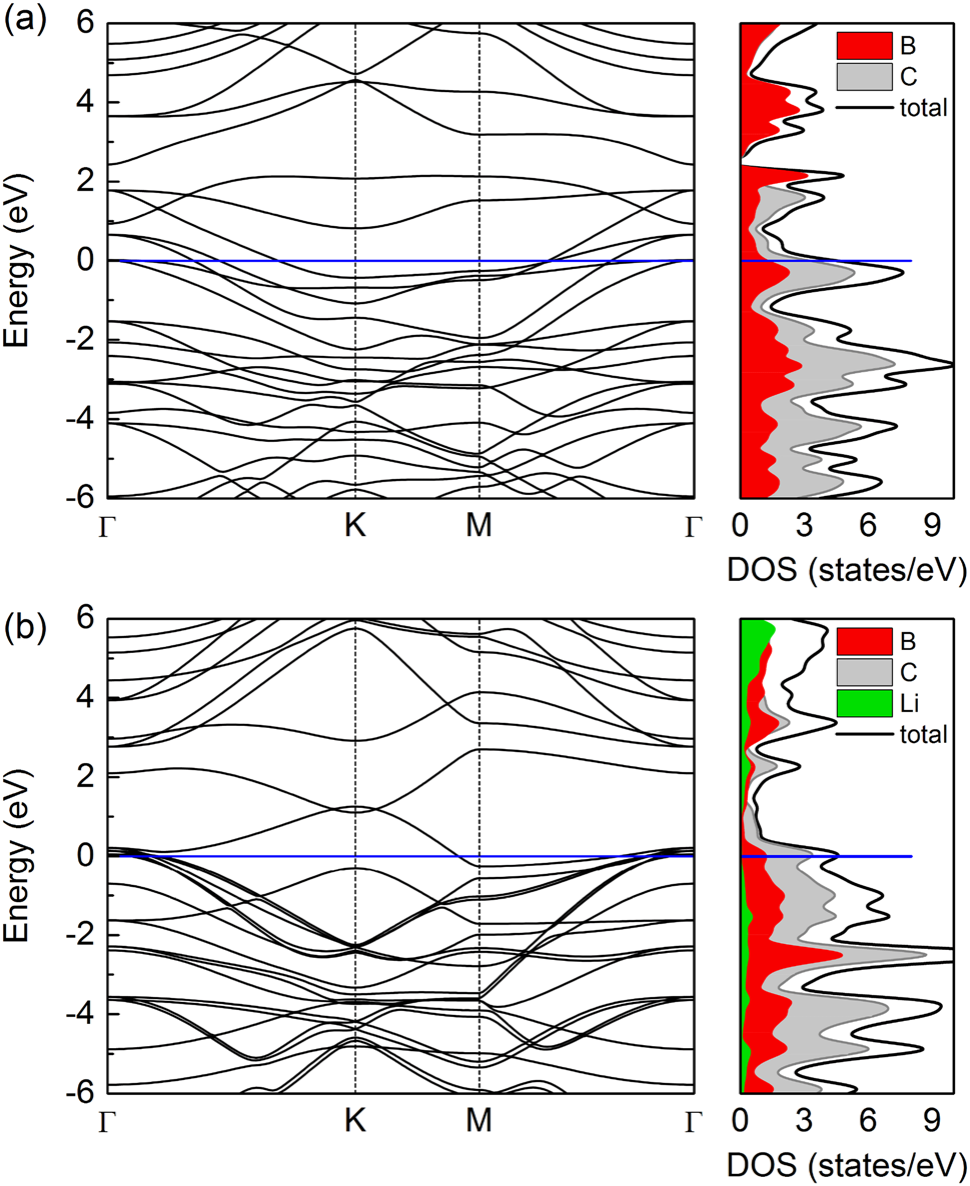
The electronic band structure along the $\Gamma$–K–M–$\Gamma$ high-symmetry line, together with the total and partial density of states for (**a**) pristine B_3_C_5_ bilayer and (**b**) Li-intercalated B_3_C_5_ bilayer.

Figure 7 represents the electronic band structure of pristine B_3_C_5_ and alterations in its electronic structure after Li intercalation. Both materials show a metallic character as indicated by the electronic states crossing the Fermi level. The metallic properties of the B_3_C_5_ bilayer offer an intrinsic profit in high electrical conductivity and a satisfying electrochemical property for better battery cycling.

**Figure 8:**
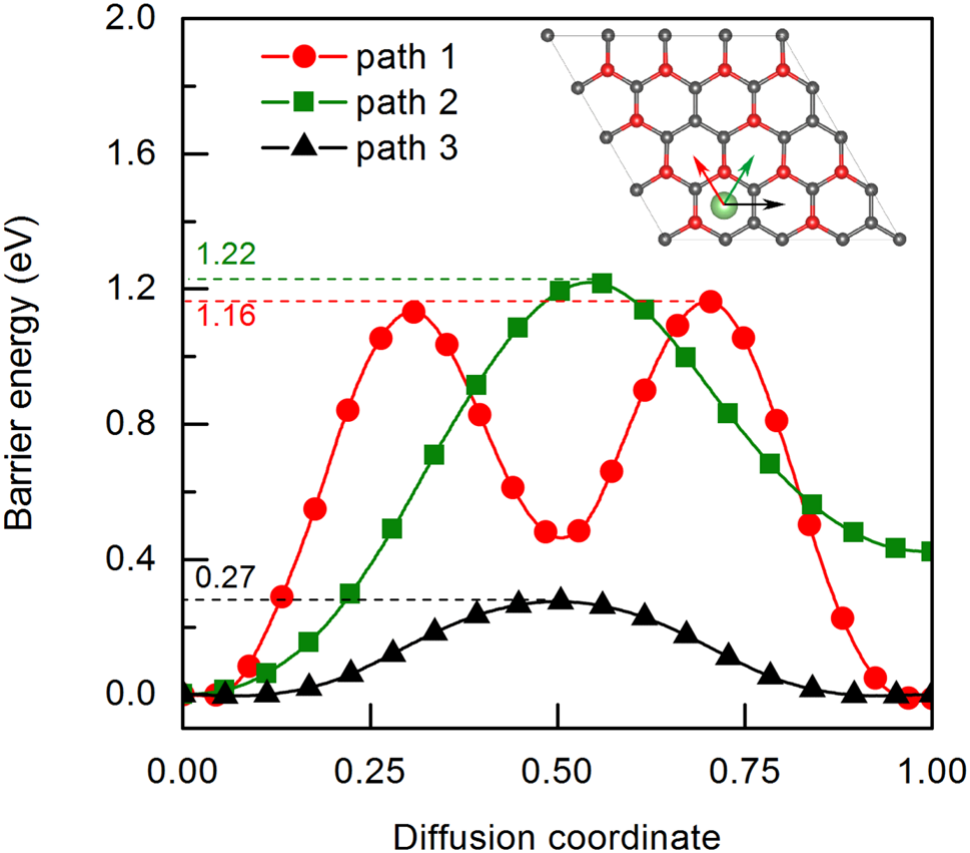
The comparison diffusion barriers in different paths of Li atom between two B_3_C_5_ layers.

To determine the diffusion and mobility features, we calculated the energy barrier for Li-ion diffusion with three main diffusion pathways as shown in Fig. 8. Path 1 is shown by the red color. The Li atom diffuses in the interlayer space from the hole of the B_2_C_4_ ring to the hole of the B_2_C_4_ ring through the C–B bridge. Path 2 is shown by the green color. The Li atom diffuses from the B_2_C_4_ ring position to the B_3_C_3_ ring also through the C–B bridge. In the case of path 3, which is shown by the black color, the Li atom migrates between two adjacent B_2_C_4_ rings through the C–C bridge. Data illustrating the diffusion stages of Li atom between two B_3_C_5_ layers are included in Supplementary Information.

The calculations reveal that the diffusion energy barriers corresponding to path 1, path 2 and path 3 are 1.16 eV, 1.22 eV and 0.27 eV, respectively. The diffusion energy barrier through the C–C bridge is lower than the C–B bridge, indicating that Li atoms are more inclined to spread in an anisotropic way across the C–C bridge. We also note that the lowest diffusion barrier obtained for the Li atom is superior to the graphene bilayer (0.34 eV)^38^, therefore, B_3_C_5_ shows great potential to serve as a LIB electrode.

The maximum theoretical storage capacity (C) is an important parameter to evaluate the performance of the LIB. It depends on the concentration of the Li ions intercalated between B_3_C_5_ layers and can be calculated using Eq. (2). The capacity of a fully lithiated B_3_C_5_ bilayer is as high as 579.57 mAh/g for LIBs (1159.15 mAh/g if we take into calculations only one layer of B_3_C_5_). The obtained result is much larger compared to the storage capacities of commercially used graphite (372 mAh/g)^6^ or TiO_2_ (335 mAh/g)^10^ and other typical 2D anode materials for LIBs like VS_2_ (466 mAh/g)^39^, Zr_2_B_2_ (526 mAh/g)^40^, NbSe_2_ (203 mAh/g)^41^, Nb_2_C (542 mAh/g)^42^, TaC_2_ (523 mAh/g)^43^ or W_2_C (259 mAh/g)^44^.

Open circuit voltage (OCV) is another crucial parameter to characterize the performance of metal-ion batteries which can be calculated using the following equation:

5 $$\begin{aligned} V\approx \frac{{-E_{\textrm{Li}_{x_2}X_3C_5}}+{E_{\textrm{Li}_{x_1}X_3C_5}}+(\mathrm {x_2-x_1})E_{\textrm{Li}}}{z(\mathrm {x_2-x_1}) {e}}, \end{aligned}$$

where, $E_{\textrm{Li}_{x_2}B_3C_5}$ and $E_{\textrm{Li}_{x_1}B_3C_5}$ are the total energies of Li-intercalated B_3_C_5_ supercell with the Li concentration of $\mathrm x_2$ and $\mathrm x_1$, respectively. The symbol *e* denotes the fundamental charge. The calculated average OCV of Li-intercalated B_3_C_5_ bilayer is 2.47 V, which is comparable to those of the commercial anode materials such as TiO_2_ with an open circuit voltage of 1.5–1.8 V^45^ and lower than OCV obtained for 2D phosphorene (2.9 V)^46^ or black arsenic (4.31 V)^47^.

## Conclusions

Lithium-ion batteries have brought about a significant transformation in the realm of energy storage, playing an indispensable role in both portable electronic devices and electric vehicles. To cater to the ever-growing demand for high-performance batteries, the quest for advanced anode materials with augmented capacity, improved lithium ion mobility, and minimal swelling effects has taken on paramount importance. This study delves into a theoretical exploration of the characteristics exhibited by novel two-dimensional B_3_C_5_ and N_3_C_5_ bilayer systems, considering their potential as anode materials for lithium-ion batteries (LIBs). The ab initio molecular dynamics (AIMD) simulations conducted at a temperature of 300 K reveal that pristine B_3_C_5_ and N_3_C_5_ bilayer systems exhibit very good stability. However, upon the introduction of lithium atoms, the N_3_C_5_ bilayer loses stability, prompting its exclusion from further analysis. Furthermore, the first-principles calculations demonstrate that the B_3_C_5_ bilayer boasts outstanding electronic conductivity, a notably lower Li diffusion barrier (0.27 V meV), and an impressive theoretical storage capacity (579.57 mAh/g). These exceptional properties strongly indicate its suitability for use as an anode material.

### Supplementary Information

Supplementary Information.

## Data Availability

The data that support the findings of this study are available in Supplementary Information.

## References

[1] MandKhan B, Chun OW, Nuengmatch P, Ullah K (2023). Role of graphene-based nanocomposites as anode material for lithium-ion batteries Mater. Sci. Eng. B.

[2] Nzereogu P, Omah A, Ezema F, Iwuoha E, Nwanya A (2022). Anode materials for lithium-ion batteries: A review. J. Appl. Surf. Sci. Adv..

[3] Xu J (2023). High-energy lithium-ion batteries: Recent progress and a promising future in applications. Energy Environ. Mater..

[4] Yuan S, Lai Q, Duan X, Wang Q (2023). Carbon-based materials as anode materials for lithium-ion batteries and lithium-ion capacitors: A review. J. Energy Storage.

[5] Salahdin OD (2022). Graphene and carbon structures and nanomaterials for energy storage. Appl. Phys. A.

[6] Nishi Y (2001). Lithium ion secondary batteries; Past 10 years and the future. J. Power Sources.

[7] Bi J (2023). On the road to the frontiers of lithium-ion batteries: A review and outlook of graphene anodes. Adv. Mater..

[8] Shi H (2022). Titanium dioxide-based anode materials for lithium-ion batteries: Structure and synthesis. RSC Adv..

[9] Yan X (2015). TiO2 nanomaterials as anode materials for lithium-ion rechargeable batteries, X. J. Energy Technol..

[10] Xia T, Zhang W, Murowchick J, Liu G, Chen X (2013). Built-in electric field-assisted surface-amorphized nanocrystals for high-rate lithium-ion battery. Nano Lett..

[11] Yadav K, Ray N (2023). Aluminene as a low-cost anode material for Li- and Na-ion batteries. J. ACS Appl. Mater. Interfaces.

[12] Durajski AP, Kasprzak GT (2023). Swelling effect of 2D BC7 anode material in Li-, Na- and Mg-ion energy storage systems. Physica B.

[13] Kasprzak GT, Szczesniak R, Durajski AP (2023). Computational insight into bilayer NC7 anode material for Li/Na/Mg-ion batteries. Comput. Mater. Sci..

[14] Bhauriyal P, Mahata A, Pathak B (2018). Graphene-like carbon-nitride monolayer: A potential anode material for Na- and K-ion batteries. J. Phys. Chem. C.

[15] Kasprzak GT, Durajski AP (2022). Two-dimensional B2C as a potential anode material for Mg-ion batteries with extremely high theoretical capacity. Sci. Rep..

[16] Durajski AP, Gruszka KM, Niegodajew P (2021). The influence of heteroatom doping on local properties of phosphorene monolayer. Sci. Rep..

[17] Wu Y, Yu Y (2019). 2D material as anode for sodium ion batteries: Recent progress and perspectives. Energy Stor. Mater..

[18] Xiao Z (2021). Recent developments of two-dimensional anode materials and their composites in lithium-ion batteries. Energy Mater..

[19] Riyanto E (2023). Lithium-ion battery performance improvement using two-dimensional materials. Today Proc..

[20] Hu Z, Liu Q, Chou S-L, Dou S-X (2021). Two-dimensional material-based heterostructures for rechargeable batteries. J. Cell Rep. Phys. Sci..

[21] Giannozzi, P. *et al*. Quantum ESPRESSO: A modular and open-source software project for quantum simulations of materials. *J. Phys. Condens. Matter* **21**, 395502 http://stacks.iop.org/0953-8984/21/i=39/a=395502 (2009). 10.1088/0953-8984/21/39/395502 21832390

[22] Giannozzi, P. *et al.* Advanced capabilities for materials modelling with Quantum ESPRESSO. *J. Phys. Condens. Matter*** 29**, 465901 http://stacks.iop.org/0953-8984/29/i=46/a=465901 (2017). 10.1088/1361-648X/aa8f79 29064822

[23] Grimme S (2006). Semiempirical GGA-type density functional constructed with a long-range dispersion correction. J. Comput. Chem..

[24] Billeter SR, Curioni A, Andreoni W (2003). Efficient linear scaling geometry optimization and transition-state search for direct wavefunction optimization schemes in density functional theory using a plane-wave basis. J. Comput. Mater. Sci..

[25] Henkelman G, Jónsson H (2000). Improved tangent estimate in the nudged elastic band method for finding minimum energy paths and saddle points. J. Chem. Phys..

[26] Kühne TD (2020). CP2K: An electronic structure and molecular dynamics software package—Quickstep: Efficient and accurate electronic structure calculations. J. Chem. Phys..

[27] KhalidButt M, MuhammadZeeshan H, AnDinh V, Zhao Y, Wang S, Jin K (2021). Monolayer SnC as anode material for Na ion batteries. Comput. Mater. Sci..

[28] Zhang Y (2017). Structural, elastic, electronic, and optical properties of the tricycle-like phosphorene. Phys. Chem. Chem. Phys..

[29] Jing Y, Ma Y, Li Y, Heine T (2017). GeP3: A small indirect band gap 2D crystal with high carrier mobility and strong interlayer quantum confinement. Nano Lett..

[30] Akgenc B (2019). New predicted two-dimensional MXenes and their structural, electronic and lattice dynamical properties. Solid State Commun..

[31] Zhao Z (2019). Metallic P3C monolayer as anode for sodium-ion batteries. J. Mater. Chem. A.

[32] Li Y, Liao Y, Chen Z (2014). Be2C monolayer with quasi-planar hexacoordinate carbons: A global minimum structure. Angew. Chem. Int. Ed..

[33] Wang D, Sun Z, Han D, Liu L, Niu L (2017). Ti3BN monolayer: the MXene-like material predicted by first-principles calculations. RSC Adv..

[34] Chen S-Y, Ye X-J, Liu C-S (2023). Monolayer BGe as a promising anode material with ultrahigh specific capacity for Mg-ion batteries. J. Phys. Lett. A.

[35] Ashuri M, He Q, Shaw LL (2016). Silicon as a potential anode material for Li-ion batteries: Where size, geometry and structure matter. Nanoscale.

[36] Chen S, Chen Z, Xu X, Cao C, Xia M, Luo Y (2018). Scalable 2D mesoporous silicon nanosheets for high-performance lithium-ion battery anode. J. Small.

[37] Zhao H (2023). Progress and perspectives on two-dimensional silicon anodes for lithium-ion batteries. ChemPhysMater.

[38] Zhong K (2019). Adsorption and ultrafast diffusion of lithium in bilayer graphene: Ab initio and kinetic Monte Carlo simulation study. Phys. Rev. B.

[39] Jing Y, Zhou Z, Cabrera CR, Chen Z (2013). Metallic VS2 monolayer: A promising 2D anode material for lithium ion batteries. J. Phys. Chem. C.

[40] Yuan G (2019). Monolayer Zr2B2: A promising two-dimensional anode material for Li-ion batteries. J. Appl. Surf. Sci..

[41] Lv X, Wei W, Sun Q, Huang B, Dai Y (2017). A first-principles study of NbSe2 monolayer as anode materials for rechargeable lithium-ion and sodium-ion batteries. J. Phys. D Appl. Phys..

[42] Hu J, Xu B, Ouyang C, Zhang Y, Yang SA (2016). Investigations on Nb2C monolayer as promising anode material for Li or non-Li ion batteries from first-principles calculations. RSC Adv..

[43] Yu T (2017). Stable and metallic two-dimensional TaC2 as an anode material for lithium-ion battery. J. Mater. Chem. A.

[44] Zhang Y (2017). First principles prediction of two-dimensional tungsten carbide (W2C) monolayer and its Li storage capability. Comput. Condens. Matter.

[45] Xia T, Zhang Y, Murowchick J, Chen X (2014). Vacuum-treated titanium dioxide nanocrystals: Optical properties, surface disorder, oxygen vacancy, and photocatalytic activities. J. Catal. Today.

[46] Li W, Yang Y, Zhang G, Zhang Y-W (2015). Ultrafast and directional diffusion of lithium in phosphorene for high-performance lithium-ion battery. Nano Lett..

[47] Akgenc B (2019). Two-dimensional black arsenic for Li-ion battery applications: A DFT study. J. Mater. Sci..

